# Influence of Device Structure and Manufacturing Thermal Budget on Channel Release Module in GAA NSFET and Process Optimization

**DOI:** 10.3390/nano16120716

**Published:** 2026-06-10

**Authors:** Meng Wang, Xinlong Guo, Ziqiang Huang, Meicheng Liao, Tao Liu, Min Xu, David Wei Zhang

**Affiliations:** 1 College of Integrated Circuits and Micro-Nano Electronics, Fudan University, Shanghai 200433, China; 22112020117@m.fudan.edu.cn (M.W.); xlguo22@m.fudan.edu.cn (X.G.); huangzq22@m.fudan.edu.cn (Z.H.); mcliao25@m.fudan.edu.cn (M.L.); dwzhang@fudan.edu.cn (D.W.Z.); 2 School of Microelectronics, Fudan University, Shanghai 200433, China

**Keywords:** gate-all-around nanosheet field effect transistor, non-plasma gas etching, channel release, nanosheet width, nanosheet spacing, thermal budget

## Abstract

In logic device development, gate-all-around nanosheet field-effect transistors (GAA NSFETs) are widely regarded as the future mainstream architecture. Due to an innovative stacked-channel design, a novel process module of channel release has been introduced, posing significant challenges to device manufacturing. The channel release quality plays a decisive role in the device’s turn-on voltage and operating speed. Meanwhile, the complex interferences are undoubtedly brought by diverse structures and manufacturing thermal budgets of GAA NSFETs. Here, the non-plasma gas etching, which is not yet widely used in the current industry, is adopted for channel release. The influences of nanosheet width, spacing, and annealing conditions on the etching process are systematically studied. A SiGe/Si etching selectivity as high as 87 is achieved. With increasing channel width, a downward trend in the single-sided damage in the central region of Si nanosheets is shown. At >100% over-etching, the Si single-sided damage in structures with different channel spacing is controlled below 1 nm. The intensified diffusion of Ge elements in the SiGe layer and a gradual slowdown of the SiGe etching rate are caused by increasing the annealing temperature. The root mean square (RMS) value of the channel surface roughness is reduced from 0.087 to 0.069 nm by adding the *H radical pretreatment into the process. These findings provide valuable guidance for developing a channel release etching process with high selectivity, low damage, a stable process window, and low fabrication difficulty.

## 1. Introduction

With the continuous scaling of transistor sizes, the FinFET structures are gradually approaching the physical limit, making it difficult to support the advanced technology nodes of 3 nm and below. Due to the strong channel control capability, the short-channel effect caused by size miniaturization is able to be effectively solved by the GAA NSFET, allowing the continuation of Moore’s Law. Therefore, the GAA NSFET is regarded as an inevitable direction for the future development of advanced semiconductor processes [1,2,3]. The channel release process module is viewed as a critical part of the fabrication process of GAA NSFET devices, which has a decisive influence on the final electrical performance of the device. The unreasonable or mismatched channel structure designs, such as channel width and spacing design, are able to induce fatal channel interface and defect problems. The imperfect process conditions, such as an insufficient etching selectivity, can lead to a degradation in the surface roughness and shape retention for the channel. So, the poor structure design or process conditions are both capable of causing a deterioration in the electrical performance of devices, finally failing to meet the actual requirements for device performance and yield [4,5,6].

The flexible, variable structural design of the GAA NSFET poses considerable challenges for the channel release process. In the FinFET device, the on-state current (I_ON_) is regulated by increasing the number of Fins. In the GAA NSFET, the difference is that the continuous adjustment of I_ON_ is achieved by changing the nanosheet width (W_NS_) at the expense of a smaller area cost [7]. Therefore, devices that meet the requirements of high performance (HP, by wide nanosheet) and low power consumption (LP, by narrow nanosheet) can be manufactured simultaneously [8,9,10]. For process development, this requires that the SiGe sacrificial layer of the wider channel device be completely removed. Meanwhile, the narrower one can withstand a large amount of over-etching, and the morphology without significant channel damage is observed. In FinFET, a higher degree of design freedom in the gate film stack is allowed because it is deposited around the Fin periphery. In contrast, the total thickness of the gate film stack in the GAA NSFET is determined by the SiGe layer thickness of the initial Si/SiGe stacked structure and is limited. Different threshold voltages (Vth) are required by transistors with diverse functions to balance overall performance and power consumption [11]. For example, in TSMC’s N2 products, the 6 different Vth are both designed in the NMOS and PMOS devices, and the regulation of Vth within a total range of 200 mV is achieved [12]. Traditionally, the fine-tuning of the device’s Vth is realized by simply adjusting the thickness of the work function metal (WFM). However, in stacked GAA NSFET devices, the WFM thickness is closely related to the nanosheet spacing. In the case of the difference in SiGe sacrificial layer thickness caused by different channel spacings, the channel release process requires that all Si channels are able to be completely released without any residual SiGe.

In addition, with the continuous shortening of gate length and the development of strain engineering, the contact resistance (R_c_) between the metal and the source/drain (S/D) is also a key factor that needs to be focused on. The reduced R_c_ is usually performed through the following approach to form ohmic contacts on strain-engineered materials in the S/D regions [13,14,15]. That is, the doping concentration of the semiconductor is adjusted to reduce the barrier width of the metal–semiconductor contact. Among them, an important regulatory role of thermal budget engineering is found. The developed process must have the ability to overcome the adverse effects caused by the thickened diffusion layer at the Si-SiGe interface, due to the diffusion of the Ge in the SiGe layer of the stacked structure being affected by all pre-layered heat-treatment processes. Therefore, there is no doubt that the SiGe/Si selective etching process in the channel release module will face a significant challenge, which is worthy of in-depth study.

The channel release module is regarded as a critical and technically challenging step in the fabrication of the GAA NSFET. It is confronted with a series of challenges, such as the strict requirements for interface state density, difficult control of etching selectivity, an extremely narrow process window, and limited thermal budget. In this work, the various test structures with different structural dimensions and manufacturing thermal budgets are designed. The effects of channel width, spacing, and annealing temperature on the etching selectivity, morphology, damage, and uniformity of the channel structure are investigated using the non-plasma gas-etching technology. The influence of annealing temperature on the diffusion of the Ge element in the Si/SiGe stacked structure and the method to improve the surface roughness of Si channels are evaluated. The basic mechanism of SiGe/Si high-selectivity etching in the channel release module is also elaborated in detail. The findings of this work provide an accurate and effective control direction for the highly selective etching process of channel release and offer ideas to the structural design and process development challenges faced in the actual manufacturing process of GAA NSFETs.

## 2. Materials and Methods

**Figure 1:**
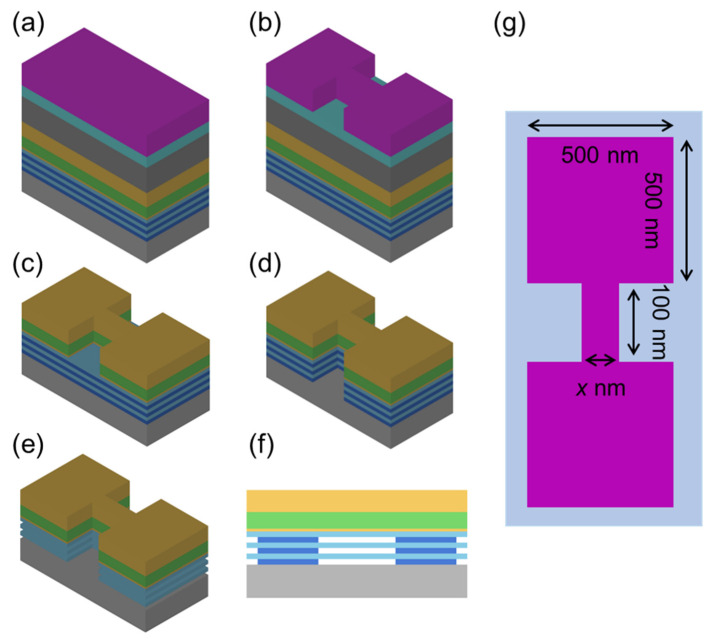
Process flow and exposure pattern of channel release module: (**a**–**f**) fabrication process, including (**a**) film deposition of Si/SiGe stacked structure and mask, (**b**) pattern lithography, (**c**) pattern transfer to ONO hard mask layer, (**d**) fin etching, (**e**) top view of channel release, (**f**) cross-section of channel structure, (**g**) pattern design of the used sample.

A relatively simple replacement structure was designed for the research and optimization of the channel release process. The specific process flow was shown in Figure 1a–f. The stack design and deposition method for the film layer of the sample were consistent with previous reports [16]. In the Si/SiGe stacked structure, the Si layer thickness of 10 nm and the Ge concentration of 25% within the SiGe layer were still maintained. In contrast, in this work, the Si channel width, SiGe sacrificial layer thickness, and heat-treatment conditions were set to the ranges of 25–80 nm, 10–25 nm, and 0–700 °C, respectively. For the exposure pattern, the structural design as shown in Figure 1g was adopted. The test pattern consisted of two large pads and a connected line in the middle of the pads. The line was used to simulate the channel, and the SiGe layer in this area was completely removed through the selective etching method to simulate the channel release process. Pads were used to simulate the S/D electrodes to provide support for the line. Only the SiGe at the edges of this area was removed during the etching process, while the majority of the interior was preserved. Specifically, the after-develop inspection critical dimension (ADI CD) of the pad and the exposure length of the line were set as 500 × 500 nm and 100 nm, and the exposure width of the line was freely adjusted within a certain range based on the mask pattern design. The non-plasma gas-etching technology was used to complete the channel release process. The etching gas system of 50 ClF_3_ + 100 He, chamber pressure of 1 Torr, and process temperature of 25 °C were set as the specific fabrication parameters. Additionally, no source and bias radio-frequency power was present in this etching system.

The morphology of the samples was characterized by transmission electron microscopy (TEM) with an operating voltage of 200 kV. The chemical composition of micro-regions in the sample was analyzed using energy-dispersive X-ray (EDX) spectroscopy equipped on the TEM. The surface roughness characterization and the acquisition of the RMS value were performed using an NX-Wafer atomic force microscope (AFM) from Park Systems. Repeated measurements were conducted in five different regions of the test sample to obtain five data points. The average and corresponding standard deviation for RMS values were then calculated.

## 3. Results and Discussion

**Figure 2:**
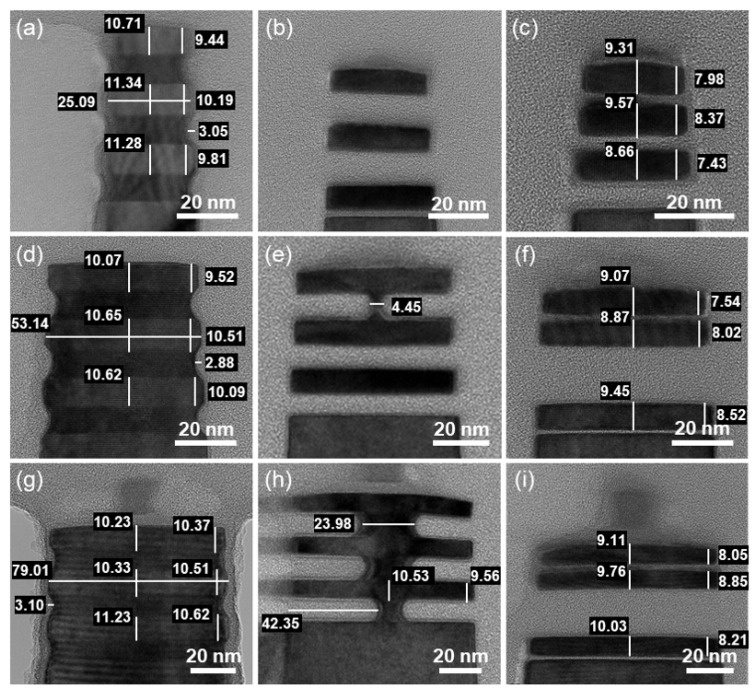
TEM images of channel release morphologies as a function of process time at W_NS_ = 25, 50, and 80 nm: (**a**–**c**) W_NS_ = 25 nm; (**d**–**f**) W_NS_ = 50 nm; (**g**–**i**) W_NS_ = 80 nm; (**a**,**d**,**g**) etching time of 10 s; (**b**,**e**,**h**) etching time of 50 s; (**c**,**f**,**i**) etching time of 100 s. The decreased Si single-sided damage from 0.97 to 0.48 nm in the nanosheet central region is discovered as the W_NS_ is increased from 25 to 80 nm, and a SiGe/Si etching selectivity as high as 87 is achieved using the chosen process.

In the GAA NSFET device, the change in channel width W_NS_ plays a crucial role in the continuous regulation of the I_ON_ [17,18,19]. In this work, test structures with a width range of 25–80 nm are designed to investigate the influence of nanosheet width on the channel release process. As shown in Figure 2, three samples with different W_NS_ of 25, 50, and 80 nm are designed, and the nanosheet thickness (T_NS_) is maintained at around 10 nm. As shown in Figure 2a,d,g, when the etching time is short (10 s), the lateral recess depth of the SiGe sacrificial layer in different-width samples is approximately the same (~3 nm). The etching time is increased to 50 s. As shown in Figure 2b,e,h, the SiGe layer of the W_NS_ = 25 nm sample is completely removed, and a square morphology at the ends of the Si layer is still observed. However, ~4 nm of residual SiGe is shown in the local area of the W_NS_ = 50 nm sample. At W_NS_ = 80 nm, more SiGe residue is found in the middle region of the channel (Figure 2h). This indicates that the etching time still needs to be extended for wider channels, but a longer over-etching time will be generated for the structure with W_NS_ = 25 nm. At this point, the SiGe/Si selective ratio of the etching process is calculated as high as 87 in accordance with the measurement results of the W_NS_ = 80 nm sample. The evaluation criteria are defined in Figure S1.

When the etching time is further increased to 100 s (Figure 2c,f,i), the SiGe sacrificial layer in the wide-channel sample is completely etched without any residual Ge element signal (Figure S2). At this point, more than 100% of the over-etching time is experienced by the narrow-channel sample with W_NS_ = 25 nm. In the three samples with W_NS_ = 25/50/80 nm, after 100 s of the etching process, 1 nm of the Si single-sided damage in the nanosheet edge region is observed, which is obviously greater than that in the center region. This is because the edge areas are continuously etched throughout the entire process, while the central areas are not exposed until the SiGe sacrificial layer is completely removed, and its etching time is relatively short. As the W_NS_ widens from 25 to 80 nm, a decreasing trend of the Si single-sided damage in the nanosheet central region is shown, decreasing from 0.97 nm to 0.48 nm. The process window for narrow-channel devices will be compressed to a certain extent when channel release processes for devices with different W_NS_ are performed simultaneously. Although the single-sided thickness of the Si channel in narrow-channel devices is damaged by about 0.5 nm more than that in wide-channel devices, the single-sided damage of less than 1 nm can still be achieved by the proposed process in this work. Usually, at the moment of channel release, the Si nanosheet channel will be subjected to certain stress changes. The length of the line designed in the test structure in this work is greater than the gate length (L_g_) of the actual GAA NSFET. Therefore, some adhesive Si layers are found in the TEM images. However, the obvious bending is not observed in the Si nanosheet, which is the key observation object, indicating that a certain degree of shape retention is possessed by the proposed process.

**Figure 3:**
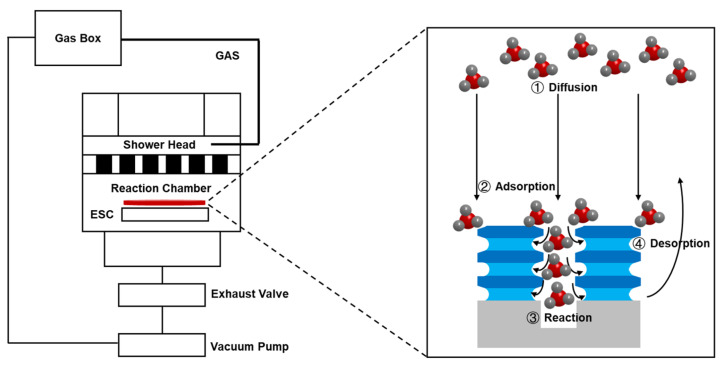
The chamber structural diagram and etching mechanism of the non-plasma gas-etching technology used in this work. The etching mechanism of the non-plasma gas technology with a high SiGe/Si selectivity mainly relies on pure thermochemical reactions, which consist of four main steps, including diffusion, adsorption, reaction, and desorption.

The SiGe/Si etching selectivity of up to 87 for the non-plasma gas-etching technique reported in this work is not only higher than that of 49 for the remote plasma source (RPS) etching system, but also higher than that of 1.3 for the inductively coupled plasma (ICP) [20,21]. This is mainly attributed to the following etching mechanism. The core of the non-plasma gas etching lies in pure thermochemical reaction, which does not have the strong bombardment ability like ICP etching technology. This etching process mainly includes the following steps, as shown in Figure 3. (1) Diffusion: gaseous etchant molecules diffuse from the top of the chamber to the wafer surface. (2) Adsorption: the etchant is adsorbed onto the sidewalls of the Si/SiGe stacked structure through physical or chemical actions. (3) Reaction: the SiGe layer undergoes a chemical reaction with the etchant molecules, generating the volatile products. In the system using ClF_3_ as the etchant, although the *F radical transfer pathway is an exothermic reaction for both Si and Ge, the activation energy of the *F transfer pathway from ClF_3_ to Ge is significantly lower than that from ClF_3_ to Si [16,22,23]. This means that the Ge-Ge bond is more prone to breakage during the fluorination reaction. Therefore, compared with Si, the SiGe sacrificial layer is easier to etch. (4) Desorption: the generated volatile products desorb from the SiGe surface and leave the reaction chamber.

**Figure 4:**
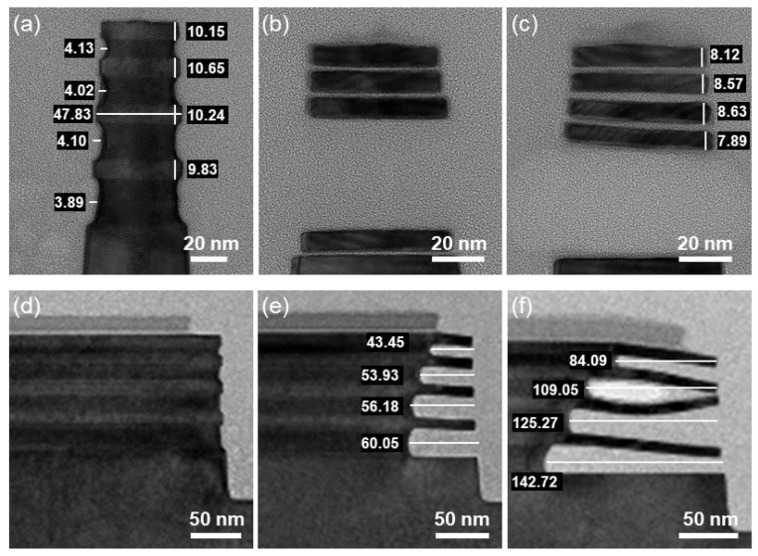
TEM images of channel release morphologies as a function of process time at different spacing T_SP_: (**a**–**c**) line area (W_NS_ = 50 nm) with (**a**) etching time of 10 s; (**b**) etching time of 50 s; (**c**) etching time of 100 s; (**d**–**f**) pad area with (**d**) etching time of 10 s; (**e**) etching time of 50 s; (**f**) etching time of 100 s. The lateral recess depth of the SiGe layer gradually increases with the enlargement of the channel spacing. At >100% over-etching, the Si single-sided damage in the structures with different channel spacing is controlled below 1 nm.

In GAA NSFETs, the filling thickness of the work function metal can be regulated by varying the nanosheet spacing (T_SP_), thereby enabling the design of multiple threshold voltages [24,25,26]. The test structures with a spacing range of 10–25 nm are designed to explore the influence of channel spacing on the channel release process, as shown in Figure 4. From top to bottom, the SiGe layer thicknesses are set at 10, 15, 20, and 25 nm, respectively (W_NS_ = 50 nm). The optimal condition described above is used to complete the etching process in this section, and the etching times of 10, 50, and 100 s are set. At a short etching time (10 s), the initial etching reactions for the SiGe layers of different thicknesses are observed. The recess depths between different layers are all maintained at 4 nm, and no obvious differences are shown (Figure 4a). At the etching time of 50 s (Figure 4b), the SiGe layers with different thicknesses are completely removed, and the EDX result is shown in Figure S3, and the square morphologies at the ends of the Si nanosheet are all observed. This experimental condition with a large process window is demonstrated by the SiGe complete removal in the stacked structure with different T_SP_. The etching time is increased to 100 s (Figure 4c). Under an over-etching of more than 100%, the single-sided damage of each Si layer from top to bottom is maintained at ~1 nm, proving that this etching process still has a high selectivity. TEM images of the pad edges are further studied to more accurately analyze the etching differences in the test structures under different T_SP_. At the etching time of 50 s (Figure 4e), the SiGe etching depths of 43.45, 53.93, 56.18, and 60.05 nm from top to bottom are observed. A significant increase in recess depth is presented when the SiGe layer thickness is greater. This is because the lower proportion of the SiGe-Si interface and the higher proportion of the bulk SiGe region are caused by the thicker SiGe layer. When etching bulk SiGe, the effects of surface effects and interface constraints on the etching reaction are suppressed, enabling a higher etching rate. Meanwhile, the reaction area is expanded by a larger thickness of the sacrificial layer, which is conducive to the diffusion of reaction gases into the interior and to the discharge of internal reaction byproducts. Conversely, the conduction of reactants and byproducts is restricted by the deep and narrow structure formed by the smaller spacing. The etching time is further increased to 100 s (Figure 4f), and the expanded difference in etching amount of SiGe layers with different thicknesses is observed. Although the difference in etching rate between the SiGe layers with different thicknesses exists, the strong controllability of the etching process is demonstrated by displaying a linear change in the etching rate.

**Figure 5:**
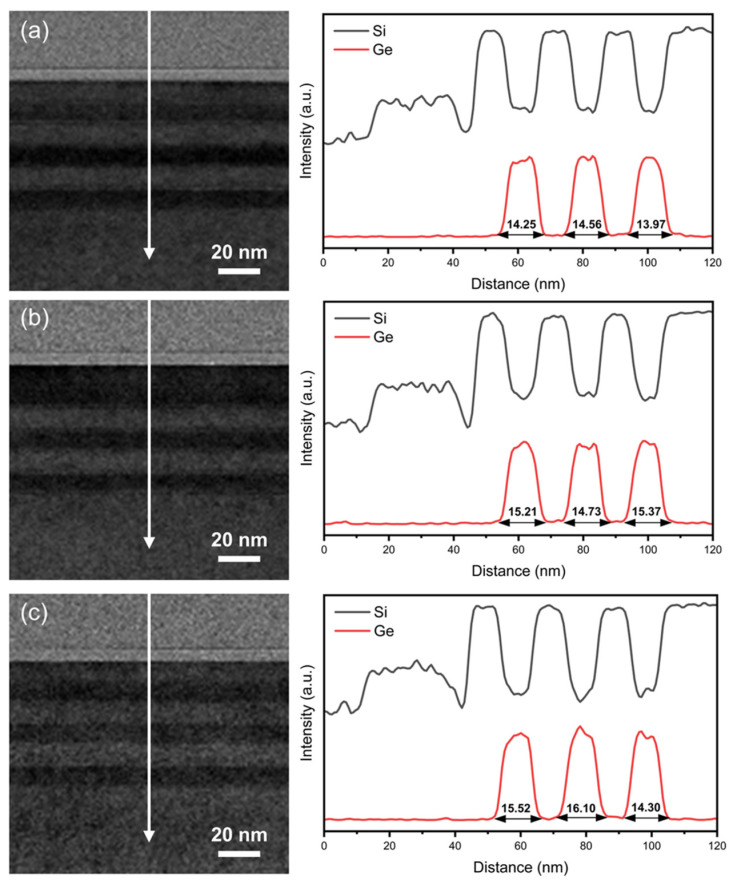
TEM images of Si/SiGe stacks and EDX elemental line scan analysis in the direction perpendicular to the film at different annealing temperatures: (**a**) unannealed; (**b**) annealing at 500 °C; (**c**) annealing at 700 °C. With increasing annealing temperature, the increased average thickness of SiGe caused by the intensified diffusion of Ge is observed from 14.25 to 15.31 nm.

To explore the influence of the manufacturing thermal budget on the diffusion of the Ge element in the Si/SiGe stacked structure, the deposited Si/SiGe test samples are annealed at 500 and 700 °C for 30 min. The element distribution of the annealed stacks is compared with that of the unannealed samples, as shown in Figure 5. In the unannealed state, the clear Si-SiGe interface in the stacked structure and the obvious contrast difference in the TEM image are observed. The average thickness of 14.25 nm for the SiGe layer is presented (Figure 5a). After annealing at 500 °C, an average SiGe thickness of 15.10 nm and a blurred Si-SiGe interface are shown (Figure 5b). After annealing at 700 °C, the average thickness of the SiGe layer increases to 15.31 nm, and the unclear contrast difference between the Si and SiGe layers is presented (Figure 5c). The contamination of Si channels results from the Ge diffusion caused by high-temperature annealing, resulting in a reduction in the pure Si thickness and the Ge content of the SiGe layer at the interface. In the channel release module of GAA NSFETs, this diffusion effect impairs not only the design thickness of the channel layer but also the roughness of the channel surface. When the low concentration of SiGe cannot be effectively removed, the residual Ge will form GeO_x_, which leads to poor channel interface properties and increased defect states, resulting in the significant degradation of the device’s subthreshold swing (SS) and I_ON_ [15,27,28].

**Figure 6:**
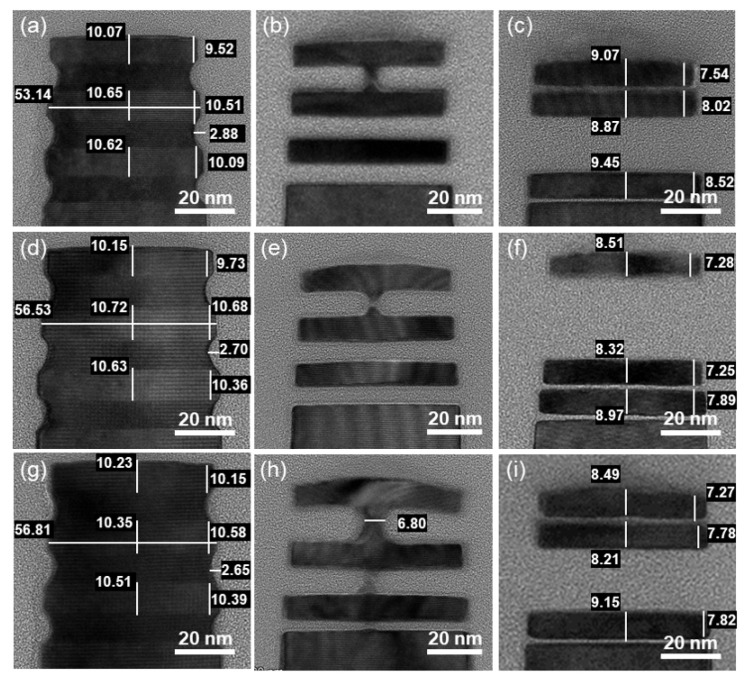
TEM images of channel release morphologies as a function of heat-treatment conditions at W_NS_ = 50 nm: (**a**–**c**) unannealed; (**d**–**f**) annealing at 500 °C; (**g**–**i**) annealing at 700 °C; (**a**,**d**,**g**) etching time of 10 s; (**b**,**e**,**h**) etching time of 50 s; (**c**,**f**,**i**) etching time of 100 s. With increasing annealing temperature, the slower SiGe etching rate is gradually presented, and the Si single-sided damage of the high-temperature annealed samples is 0.4 nm greater than that of the unannealed ones.

The influence of the manufacturing thermal budget on the channel release process is further investigated, as shown in Figure 6 (W_NS_ = 50 nm). At the etching time of 10 s, the recess depth of ~3 nm for the SiGe layer in different samples is observed. When the etching time is increased to 50 s, the SiGe layer of the unannealed and 500 °C annealed samples is almost removed, but 6.8 nm of the residual SiGe in the local area of the 700 °C sample is found. It is speculated that the slowed etching rate of the SiGe layer under the high-temperature annealing conditions is caused by the reduction in Ge concentration, the increase in interface defects, and the intensification of lattice mismatch [29,30]. At the etching time of 100 s, the full channel release of the different samples is completed, and the over-etching of 100% in process is obtained to ensure complete channel release. In addition, compared with the central region, the greater damage on the edge of the nanosheets is still suffered due to prolonged exposure to the etching atmosphere. Overall, the Si single-sided damage of the high-temperature annealed samples is 0.4 nm greater than that of the unannealed ones, which is caused by the loss of the high-temperature annealing on the etching selectivity. The EDX mapping images of the channel release process with different heat-treatment conditions are shown in Figure S4, and no obvious Ge signal is observed in the etched area. The excellent performance of the non-plasma gas-etching process proposed by this work is demonstrated in terms of realizing channel release under high-temperature annealing, and has an important reference value for the actual manufacturing of the GAA NSFET. The topic of further understanding the etching behavior and mechanism by the electrical characteristic measurement of the fabricated samples will be explored in the subsequent work.

**Figure 7:**
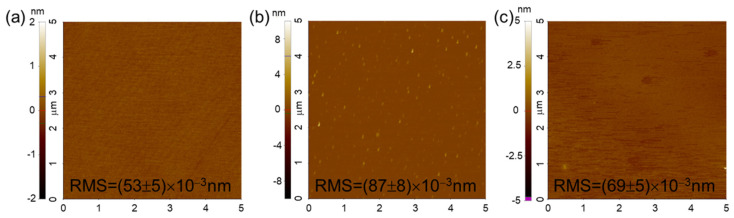
The influence of a non-plasma gas-etching process on the surface roughness of the channel structure: (**a**) before the etching process; (**b**) pretreatment without *H radicals; (**c**) after *H radical pretreatment. The Si surface damage caused by the etching process is improved by *H free radical pretreatment, and the reduced RMS value from (87 ± 8) × 10^−3^ to (69 ± 5) × 10^−3^ nm is obtained.

In the actual manufacturing of the GAA NSFET, not only is the damage to the channel thickness considered by the channel release module, but also the increase in the channel surface roughness. A reduction in the electrical performance of the device results from the influence of the interface property on surface roughness. The effect of the non-plasma gas-etching process on the channel surface roughness is investigated. As shown in Figure 7b, the enlarged RMS value of the Si surface roughness from (53 ± 5) × 10^−3^ to (87 ± 8) × 10^−3^ nm is observed after the non-plasma gas etching. Inspired by the fact that the reduction in chemical potential is caused by the ability of *H free radicals to repair Si dangling bonds [31], an improved process scheme is proposed. That is, the *H pretreatment process is introduced. The entire gas-etching process is divided into multiple cycles, each of which is composed of two processes, including *H free-radical pretreatment of 15 s and ClF_3_-based non-plasma gas etching of 20 s. As shown in Figure 7c, the surface roughness of the sample is improved to some extent, and the reduced RMS value from (87 ± 8) × 10^−3^ to (69 ± 5) × 10^−3^ nm is obtained. This demonstrates that the introduction of *H radical pretreatment is conducive to mitigating the Si channel surface damage caused by the etching process.

## 4. Conclusions

The channel release module of the GAA NSFET device is the focus of this work. The influences of channel width, channel spacing, and manufacturing thermal budget on the etching process behavior are investigated in detail. A non-plasma gas-etching process scheme with high SiGe/Si selectivity, low Si damage, and a stable window is proposed. The decreased Si single-sided damage from 0.97 to 0.48 nm in the nanosheet central region is discovered as the W_NS_ is increased from 25 to 80 nm. A SiGe/Si etching selectivity as high as 87 is achieved using the chosen process. The lateral recess depth of the SiGe layer gradually increases with the enlargement of the channel spacing. At >100% over-etching, the Si single-sided damage in the structures with different channel spacing is controlled below 1 nm. With increasing annealing temperature, the increased average thickness of SiGe caused by the intensified diffusion of Ge element is observed from 14.25 to 15.31 nm, and the slower SiGe etching rate is gradually presented. The Si surface damage caused by the etching process is improved by *H free-radical pretreatment. This work provides reliable and important insights into the structural/thermal budget design and process development for the channel release module in the GAA NSFET.

## Data Availability

Data presented in this study are available on request from the corresponding author.

## Supplementary Materials

The following supporting information can be downloaded at https://www.mdpi.com/article/10.3390/nano16120716/s1. Figure S1: The calculation formulas for the SiGe/Si etching selectivity, the Si single-sided loss, and the structural diagrams. Figure S2: TEM and EDX mapping images of the channel release etching at different W_NS_: (a) W_NS_ = 25 nm; (b) W_NS_ = 80 nm. Figure S3: TEM and EDX mapping images of the channel structure with different spacing T_SP_ as a function of process time at W_NS_ = 50 nm: (a) etching time of 50 s; (b) etching time of 100 s. Figure S4: TEM and EDX mapping images of the channel release process with different heat-treatment conditions at W_NS_ = 50 nm: (a) annealing at 500 °C; (b) annealing at 700 °C.
