# Supplementary material for: Influence of Device Structure and Manufacturing Thermal Budget on Channel Release Module in GAA NSFET and Process Optimization
Source: Nanomaterials (Basel). 2026 Jun 10;16(12):716. doi: 10.3390/nano16120716 (PMC13304493; doi:10.3390/nano16120716)
Supplement: Supplementary file 1 [file nanomaterials-16-00716-s001.zip › nanomaterials-4315142-supplementary.pdf]

nanomaterials

Article

Influence of Device Structure and Manufacturing Thermal Budget on Channel Release Module in GAA NSFET and Process Optimization

Meng Wang [1,2], Xinlong Guo [1,2], Ziqiang Huang [1,2], Meicheng Liao [1,2], Tao Liu [1,2,*], Min Xu [1,2,*], and David Wei Zhang [1,2]

[1] College of Integrated Circuits and Micro-Nano Electronics, Fudan University, Shanghai 200433, China
[2] School of Microelectronics, Fudan University, Shanghai 200433, China
* Correspondence: tliu14@fudan.edu.cn (T.L.); xu_min@fudan.edu.cn (M.X.)

Supplementary Materials

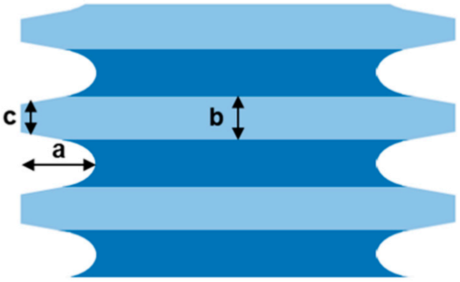

$$Si\ loss@each\ side = \frac{b-c}{2}$$

$$Selectivity = \frac{a}{(b-c)/2}$$

Figure S1. The calculation formulas for the SiGe/Si etching selectivity, the Si single-sided loss, and the structural diagrams.

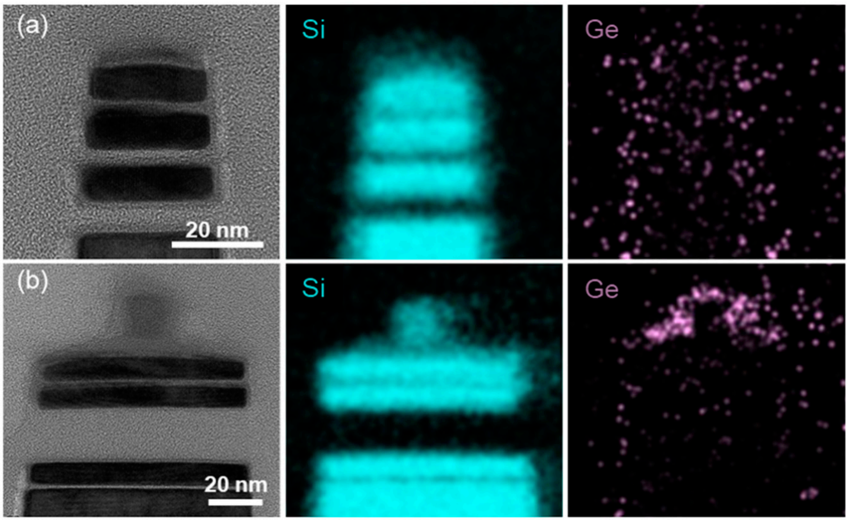

Figure S2. TEM and EDX mapping images of the channel release etching at different W_{NS}: (a) W_{NS} = 25 nm; (b) W_{NS} = 80 nm.

 https://doi.org/10.3390/nano16120716

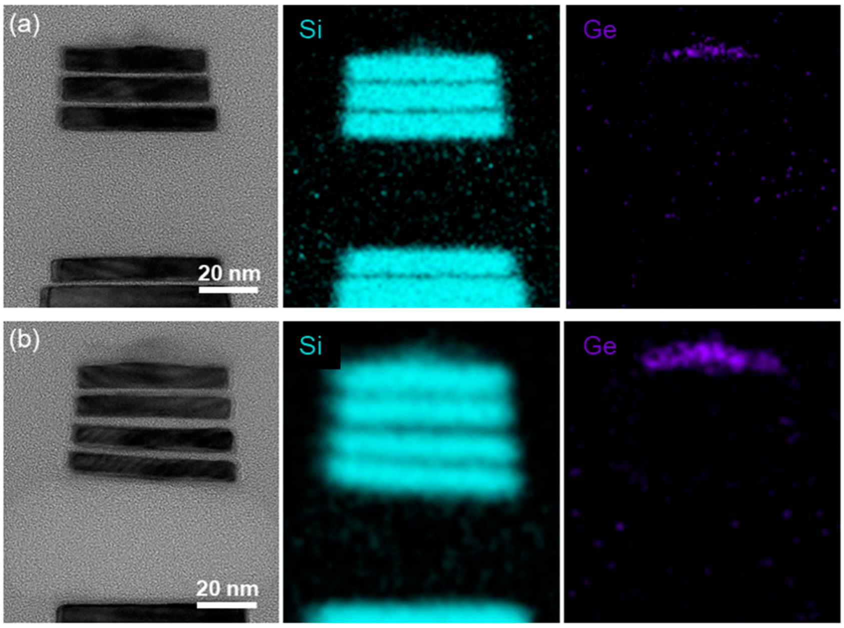

Figure S3. TEM and EDX mapping images of the channel structure with different spacing T_{SP} as a function of process time at W_{NS} = 50 nm: (a) etching time 50 s; (b) etching time 100 s.

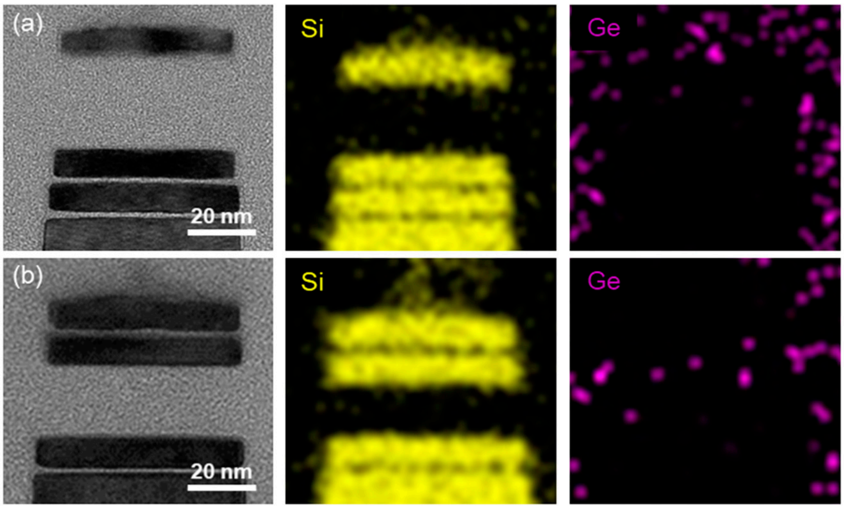

Figure S4. TEM and EDX mapping images of the channel release process with different heat treatment conditions at W_{NS} = 50 nm: (a) annealing at 500 °C; (b) annealing at 700 °C.

https://doi.org/10.3390/nano16120716
